# Potential quality indicators for seriously ill home care clients: a cross-sectional analysis using Resident Assessment Instrument for Home Care (RAI-HC) data for Ontario

**DOI:** 10.1186/s12904-018-0389-y

**Published:** 2019-01-09

**Authors:** Lisa E. Harman, Dawn M. Guthrie, Joachim Cohen, Anja Declercq, Kathryn Fisher, Donna Goodridge, John P. Hirdes, Hsien Seow

**Affiliations:** 1 Department of Kinesiology and Physical Education, Wilfrid Laurier University, 75 University Avenue West, Waterloo, Ontario N2L 3C5 Canada; 2 Department of Health Sciences, Wilfrid Laurier University, 75 University Avenue West, Waterloo, Ontario N2L 3C5 Canada; 3 End-of-Life Care Research Group, Faculty of Medicine, Vrije Universiteit Brussel, Laarbeeklaan 103, 1090 Brussels, Belgium; 4 LUCAS-Centre for Care Research and Consultancy, K.U. Leuven, Minderbroedersstraat 8 - box 5310, 3000 Leuven, Brussels Belgium; 5 School of Nursing, McMaster University, HSC Room 3N25G, 1280 Main St West, Hamilton, Ontario L8S 4K1 Canada; 6 College of Medicine, University of Saskatchewan, Room 543 Ellis Hall, 108 Hospital Drive, Saskatoon, Saskatchewan S7N 0W8 Canada; 7 School of Public Health & Health Systems, University of Waterloo, 200 University Avenue West, Waterloo, Ontario N2L 3G1 Canada; 8 Juravinski Cancer Centre, 699 Concession Street, 4th Floor, Room 4-229, Hamilton, Ontario L8V 5C2 Canada

**Keywords:** Quality indicators, Quality measures, Palliative care, Seriously ill, InterRAI, End-of-life care, Home care

## Abstract

**Background:**

Currently, there are no formalized measures for the quality of home based palliative care in Ontario. This study developed a set of potential quality indicators for seriously ill home care clients using a standardized assessment.

**Methods:**

Secondary analysis of Resident Assessment Instrument for Home Care data for Ontario completed between 2006 and 2013 was used to develop quality indicators (QIs) thought to be relevant to the needs of older (65+) seriously ill clients. QIs were developed through a review of the literature and consultation with subject matter experts in palliative care. Serious illness was defined as a prognosis of less than 6 months to live or the presence of severe health instability. The rates of the QIs were stratified across Ontario’s geographic regions, and across four common life-limiting illnesses to observe variation.

**Results:**

Within the sample, 14,312 clients were considered to be seriously ill and were more likely to experience negative health outcomes such as cognitive performance (OR = 2.77; 95% CI: 2.66–2.89) and pain (OR = 1.59; 95% CI: 1.53–1.64). Twenty subject matter experts were consulted and a list of seven QIs was developed. Indicators with the highest overall rates were prevalence of falls (50%) prevalence of daily pain (47%), and prevalence of caregiver distress (42%). The range in QI rates was largest across regions for prevalence of caregiver distress (21.5%), the prevalence of falls (16.6%), and the prevalence of social isolation (13.7%). Those with some form of dementia were most likely to have a caregiver that was distressed (52.6%) or to experience a fall (53.3%).

**Conclusion:**

Home care clients in Ontario who are seriously ill are experiencing high rates of negative health outcomes, many of which are amenable to change. The RAI-HC can be a useful tool in identifying these clients in order to better understand their needs and abilities. These results contribute significantly to the process of creating and validating a standardized set of QIs that can be generated by organizations using the RAI-HC as part of normal clinical practice.

## Background

The main goals of palliative care are to address the physical, social, psychological, and spiritual needs of an individual with serious-illness and to support their family and other informal caregivers during the dying process [1, 2]. Increasingly, research has focused on home-based palliative care for a number of reasons. Most patients with life-limiting illnesses prefer to receive care at home [3], which increases the likelihood that a person will die at home if that was their preference [4]. In addition, home-based care has been shown to improve patient satisfaction and lower costs when compared with long-term care or hospital-based care [4]. Individuals who are considered to be seriously ill (SI) include persons with life-limiting diagnoses such as those with terminal cancer, organ failure, and Alzheimer’s dementia/other dementias and/or individuals with a short prognosis (i.e., typically less than 6 months). It is increasingly recognized that these individuals could benefit from a palliative approach to care [5]. Therefore, end-of-life care should be relevant and appropriate for those with different types of serious illness. However, few formalized methods exist for evaluating the *quality* of home-based care. Research has instead focused on the presence of home-based care services, their utilization, organization and the cost-effectiveness of these services [6, 7]. Therefore establishing a formalized method, such as a set of standardized quality indicators (QIs) for home-based care for seriously ill clients is essential.

QIs are expressed as rates within a population and measure important health outcomes to allow for comparisons between different health care settings, providers, or geographic regions. QIs flag potential issues that can support continuous quality improvement initiatives. A recent update of a systematic review of existing QIs for palliative care identified 29 relevant papers that demonstrated indicators exist in multiple countries and capture the relevant domain areas for palliative care [8]. For example QIs for palliative care have been developed in the USA, United Kingdom, Italy and Japan. More recent development activities are also demonstrated in other countries such Belgium [9, 10]. In Canada, a small number of studies have proposed potential QIs focusing mainly on administrative data (e.g., hospital admissions, emergency room visits) for cancer patients [11, 12], but not for non-cancer patients, who can also benefit from palliative care services [13]. Therefore, despite some early work in Canada, there are no standardized QIs for palliative care services that could be reported at a local, provincial, or national level.

To address the absence of a set of Canadian QIs, the main objective of the current study was to develop a set of QIs for seriously ill home care clients based on items within the Resident Assessment Instrument for Home Care (RAI-HC), a standardized assessment used in Ontario [14]. InterRAI assessments have previously been used to create QIs for long-term care [15], the general home care population [16], post-acute care [17], and for inpatient psychiatry [18]. These assessments are valid instruments that are used in multiple parts of Canada and internationally [19–21], but currently have not been used to create QIs for seriously ill home care clients. A secondary objective was to examine the rates of these preliminary QIs for specific life-limiting diagnoses and also for each of the 14 Local Health Integration Networks (LHINs) in Ontario. These LHINs represent a distinct geographic region of Ontario and are responsible for providing health care services within that region.

## Methods

### Sample

This project represents a cross-sectional secondary analysis of RAI-HC data collected for home care clients aged 65+ and who were seriously ill in Ontario. Each year roughly 700,000 individuals receive publicly-funded home care in this province [22] and approximately 250,000 new RAI-HC assessments are completed annually. The RAI-HC data were obtained from the Canadian Institute for Health Information. The overall sample represented 417,273 individuals assessed with the RAI-HC between 2006 and 2013. The analysis was restricted to individuals who had at least two RAI-HC assessments and had been receiving home care for at least 30 days. This was done to allow for an adequate period of time for the home care team to intervene and have an influence on the person’s health status that would be reflected in the QI rates, therefore creating a fair assessment of the outcomes of care provided. Eligible assessments were the client’s most recent assessment that were not completed on admission into the home care program. After exclusions, 264,217 unique individuals remained in the analytic sample and were divided into two mutually exclusive groups, namely, those who were seriously ill and not seriously ill. Those in the seriously ill group had a prognosis of less than 6 months (as indicated by a single dichotomous item on the RAI-HC), or had a CHESS score of four or higher, which represents a high level of health instability and is associated with a lower probability of survival [23–25]. The CHESS scale (Changes in Health, End-stage Disease, Signs and Symptoms) is a health index scale embedded within the RAI-HC that captures a person’s level of health instability and risk of mortality. All remaining clients were categorized as not seriously ill.

In Ontario, home care clients are typically designated as being “palliative” using an administrative code when they become part of the palliative care caseload. However, this information was unavailable within the current dataset so the clients included in our dataset may or may not have been receiving formal palliative services.

### Measures

InterRAI is a non-profit network of about 100 researchers and clinicians from over 35 countries who develop, test, and validate assessment tools for a variety of health care settings [21]. The RAI-HC includes roughly 300 items capturing domains such as cognition, communication, mental health and physical functioning [26] and was mandated in 2002 by the Ontario government for use with all long-stay clients (i.e., those expecting to receive home care for at least 60 days) [25]. The RAI-HC is currently mandated or used voluntarily in eight Canadian provinces/territories and in many other countries. The assessment is completed using a laptop in the client’s home by a trained professional (typically a registered nurse) and is repeated in increments of approximately 6 months, unless a change in health status warrants an earlier re-assessment [27]. The electronic assessments are stored within a pan-Canadian database held by the Canadian Institute for Health Information where they can be accessed by researchers and students. Previous research has established the validity and reliability of measures within the RAI-HC [14, 21, 26, 28]. For example, convergent validity has been established for multiple health index scales embedded within the instrument [26] and individual items have been shown to have good inter-rater reliability (mean kappa = 0.69) [21, 26].

The health index scales within the RAI-HC provide important information about the functional status and well-being for each client [29]. The scales are generated electronically and available as a summary report to the assessor. For example, the Instrumental Activities of Daily Living (IADL) scale is a summative score across seven items that ranges from 0 to 21. These items encompass activities such as housework and meal preparation where a higher score indicates greater impairment [30]. The Activities of Daily Living Self-performance Hierarchy Scale (ADL-S) ranges from zero (completely independent) to six (total dependence) [31]. This scale is hierarchical and assigns higher weights to late-loss ADLs (e.g., eating, locomotion) and lower weights to early-loss ADLs (e.g., hygiene, toilet use). Both the IADL scale and the ADL-S are reliable and valid measures of functional performance [21, 32].

The Depression Rating Scale (DRS) indicates signs and symptoms of depression. It is a summative score across seven items with a maximum score of 14. A score of three or higher has been shown to accurately predict the development of a clinical diagnosis of depression [20, 33–35]. The Cognitive Performance Scale (CPS) is scored from zero to six and is based on four items including short-term memory, expressive communication, independence in eating, and decision-making. A higher score indicates a greater degree of impairment [32, 36]. The scale has established criterion validity when compared to the Mini-Mental State Examination [32]. The Pain Scale [37] is comprised of two items: pain frequency and pain intensity and is scored from zero (no pain) to three, which indicates pain that is daily and severe/excruciating. Criterion validity has been established against the Visual Analogue Scale [38]. The CHESSCHESS scale is comprised of 12 items including changes in ADL status, cognition, shortness of breath, and a prognosis of less than 6 months to live [24]. The CHESS scale is scored from zero to five, with higher scores indicating greater health instability. It is a strong predictor of mortality, regardless of the individual’s demographic characteristics and medical diagnoses. For each one-point increase on the CHESS scale, an individual has nearly a two-fold increased risk of death [23].

### Development of preliminary Quality Indicators (QIs)

We conducted a comprehensive literature review regarding the key domains to capture when assessing quality in palliative care and also reviewed the existing interRAI QIs for home care and long-term care [15, 16]. Following this, the team developed a potential list of 17 QIs that could be operationalized using items within the RAI-HC. We excluded QIs if items used to generate the QI overlapped with the inclusion criteria for the seriously ill group (i.e., items within the CHESS scale such as dehydration and dyspnea). The list of 17 indicators was then modified through consultations with 21 members of an expert panel. Prior to each meeting with the experts, they were given a list of the 17 potential indicators along with the definition for the numerator and denominator for each QI. The experts were identified through consultation with members of the research team and included individuals from Canada (Ontario, Alberta, Manitoba), the United States, and Belgium. Front-line clinicians (*n* = 6; e.g., nurses, palliative care physicians) made up roughly 29% of the sample, another six (29%) were academic researchers, and the remaining nine, were care managers or clinical directors.

The experts were asked about each of the 17 preliminary QIs and provided suggestions for improving or modifying each indicator or suggested new indicators, where appropriate. Potential QIs were kept for further consideration if the majority of experts felt it was important, if the literature substantiated the importance of assessing the particular issue or outcome when measuring quality and if the particular QI could be generated using data elements within the RAI-HC. The experts also suggested appropriate client-level risk adjusters for one QI, namely, the prevalence of falls. Risk adjustment is a useful statistical technique that attempts to adjust for differences in patient acuity when comparing QIs across different providers or across geographic boundaries considering logistic regression models [39–41].

### Analysis

The two main groups (i.e., seriously ill and not seriously ill) were compared across demographic characteristics, the health index scales, several diagnoses (e.g., cancer, congestive heart failure, chronic obstructive pulmonary disease/asthma/emphysema [a single item on the RAI-HC], Alzheimer’s dementia (AD) and other types of dementia), physical functioning and mental status. All diagnoses were measured as dichotomous variables. This analysis was completed to establish differences between the two groups and verify that the seriously ill group was in worse health than the comparison group.

The large size of the analytic sample (*n* = 264,217) would have resulted in an inflated probability of making a type I error due to small *p*-values. We therefore chose to use odds ratios as an indication of clinical significance instead of relying strictly on p-values. Odds ratios were deemed clinically significant if they represented a change in the odds of the event between the SI and not-seriously ill groups of at least 20% (i.e., odds ratio of ≥1.20 or inversely ≤0.83). This cut-point was chosen based on previous studies of pain and depression that have suggested a clinically relevant difference ranging from 13 to 16% [42, 43]. All QI rates were expressed as a percentage. The rates were calculated for the province as a whole and also for each of the 14 health regions.

Suggestions from the expert panel, as well as evidence from analysis of the RAI-HC data, were taken into account when deciding which client-level covariates would be used in the risk adjustment process for the falls QI [40, 44, 45]. Potential covariates were entered first individually, and then using several stepwise procedures, into a binary logistic regression model with the individual QI scores (presence or absence) as dependent variables. Only covariates with an odds ratio of ≥1.30, or inversely, ≤0.77, were kept as risk adjusters, in line with previous research [45]. This study has been approved by Wilfrid Laurier University’s Research Ethics Board (REB # 6003004).

## Results

A total of 14,312 individuals (5.4%) were considered to be seriously ill, and within this group, 16.9% had only a prognosis of 6 months or less, 65.3% had only a score of four or higher on the CHESS scale, and 17.8% met both criteria. The remaining 249,905 (94.6%) individuals were considered as the comparison group who ere not seriously ill. The two groups were very similar on all demographic characteristics and all absolute differences were very small (Table 1). The odds ratios exceeded the 20% cut-point when comparing the two groups across all of the health index scales, and always in the direction of the seriously ill group being more likely to experience impairments or limitations. For example, 79% of the seriously ill group had moderate to severe cognitive impairment compared to 57.6% in the comparison group, a nearly three-fold increase in the odds (Table 1). Seriously ill individuals were also significantly more likely to have a life-limiting diagnosis, such as cancer (odds ratio = 3.09; 95% CI: 2.97–3.20), congestive heart failure (odds ratio = 1.88; 95% CI: 1.81–1.96), or chronic obstructive pulmonary disease/asthma/emphysema [a single item on the RAI-HC], odds ratio = 1.62; 95% CI: 1.56–1.68) (Table 1).

**Table 1:** Demographic health-related outcomes comparing clients who are seriously ill and not seriously ill

Characteristic	Not seriously ill (NSI) *N* = 249,905	Seriously ill (SI) *N* = 14,312	Univariate odds ratio comparing SI vs. NSI (95% CI ^a^)
	% (*n*)		
Age			
65–74	12.9 (32321)	13.1 (1868)	Ref
75–84	39.1 (97814)	38.1 (5452)	0.96 (0.95–1.02)
85+	47.9 (119770)	48.9 (6992)	1.01 (0.96–1.07)
Sex			
Male	34.2 (85438)	38.0 (5434)	Ref
Female	65.8 (164467)	62.0 (8878)	0.85 (0.82–0.88)
Diagnoses			
Cancer	13.2 (33072)	32.0 (4580)	3.09 (2.97–3.20)
Congestive heart failure (CHF)	15.4 (38479)	25.6 (3657)	1.88 (1.81–1.96)
Chronic obstructive pulmonary disease (COPD)	19.0 (47423)	27.5 (3934)	1.62 (1.56–1.68)
Alzheimer’s disease or other dementia	31.3 (78,206)	34.9 (4991)	1.20 (1.12–1.24)
Depression Rating Scale			
No signs/symptoms of depression (0–2)	81.0 (202513)	62.7 (8968)	Ref
Signs/ symptoms of depression (3–14)	19.0 (47392)	37.3 (5344)	2.55 (2.46–2.64)
Cognitive Performance Scale			
Intact/ Mild Impairment (0–1)	42.4 (105928)	21.0 (3005)	Ref
Moderate/ Severe Impairment (2–6)	57.6 (143933)	79.0 (11306)	2.77 (2.66–2.89)
Pain Scale			
No pain/less than daily pain (0–1)	46.1 (115252)	35.0 (5014)	Ref
Daily/severe pain (2, 3)	53.9 (134644)	65.0 (9298)	1.59 (1.53–1.64)
Activities of Daily Living Self-performance Hierarchy Scale			
No/mild impairment (0–1)	79.5 (198701)	57.5 (8228)	Ref
Moderate/severe impairment (2–6)	20.5 (146320)	42.5 (6084)	2.87 (2.77–2.97)
Instrumental Activities of Daily Living Inventory Scale			
No/mild impairment (0–13)	41.5 (103575)	19.4 (2771)	Ref
Moderate/severe impairment (14–21)	58.6 (146320)	80.6 (11541)	2.95 (2.83–3.08)
Marital status			
Never married	4.3 (10783)	2.9 (415)	Ref
Married/other	36.7 (91638)	39.0 (5588)	1.58 (1.43–1.75)
Widowed/ separated/divorced	59.0 (147484)	58.1 (8309)	1.46 (1.32–1.62)
Education level			
Completed grade 11 or less	61.1 (152758)	62.7 (8966)	Ref
High school	17.0 (42384)	16.4 (2340)	0.94 (0.90–0.99)
Post-secondary	21.9 (54748)	21.0 (3005)	0.94 (0.90–0.98)
Language			
English	78.5 (196117)	81.0 (11592)	Ref
French	3.0 (7559)	3.1 (445)	1.0 (0.90–1.10)
Other	18.5 (46229)	15.9 (2275)	0.83 (0.78–0.87)

^a^ *CI* confidence interval

A list of the seven preliminary QIs, their operational definitions, and the overall rate for Ontario is shown in Table 2. Other possible QIs were dropped from further consideration including failure to achieve a sense of life completion, breakthrough pain, fatigue and sleep problems. These QIs were removed from further analysis since the RAI-HC did not include the items need to generate the QI. Two other potential indicators, namely dehydration and dyspnea, were excluded since these items are captured in the CHESS scale which was used to identify our sample of seriously ill clients.

**Table 2:** Quality Indicator operational definitions and mean rate in the seriously ill group

Quality indicator	Mean QI rate (*N* = 14,312) % (*n*)	Operational definition	
		Numerator	Denominator
Prevalence of Falls	49.0 ^a^ (6411)	Clients who record a fall on a follow up assessment	Those not completely dependent on bed mobility Risk Adjusters: Parkinson’s disease, ADL impairment, vision impairment
Prevalence of Disruptive or Intense Daily Pain	46.6 (6664)	Client is having daily pain -AND- It is severe or excruciating pain	All clients on re-assessment
Prevalence of Caregiver Distress	41.7 (5909)	Client’s primary caregiver experiences feelings of distress, anger or depression	All clients on reassessment who have a primary caregiver
Prevalence of Negative Mood	26.9 (3847)	Feeling of sadness or bring depressed) -AND- -at least two of: persistent anger, repetitive health complaints, sad, pained, worried facial expressions, recurrent crying, tearfulness, withdrawal from activities, reduced social interaction	All clients on re-assessment
Prevalence of Inadequate Medication to Control Pain	22.8 (2875)	Client has pain -AND- Medications do not adequately control pain	All clients on reassessment who experience pain
Prevalence of Social Isolation	21.1 (3021)	Client is alone for long periods of time or all of the time - AND - Client indicates feeling lonely -OR- Decline in social activities, client is distressed	All clients on re-assessment
Prevalence of Constipation	3.5 (499)	No bowel movement in 3 days	All clients on re-assessment

^a^ adjusted rate

The highest overall rates were seen for falls (49.0%), disruptive or intense daily pain (46.6%) and caregiver distress (41.7%). Some of the QIs displayed considerable variation between the regions. For example, when comparing the regions with the highest and lowest rates across the QIs, the largest difference was for caregiver distress (range = 21.5%), followed by falls (range = 16.6%), social isolation (range = 13.7%) and negative mood (range = 12.1%) (Table 3). The falls QI was adjusted for the CPS score (OR = 2.20), ADL-S score (OR = 1.31), the presence of vision impairment (OR = 1.30), and a diagnosis of Parkinson’s disease (OR = 1.85). The adjusted rate for falls is presented in Table 3.

**Table 3:** Prevalence rates for the seven quality indicators by geographic region

Quality indicator	Geographic region														
	1	2	3	4	5	6	7	8	9	10	11	12	13	14	Range ^a^
	*n* = 599	*n* = 1635	*n* = 758	*n* = 1821	*n* = 349	*n* = 578	*n* = 599	*n* = 1780	*n* = 1561	*n* = 929	*n* = 1228	*n* = 1175	*n* = 907	*n* = 353	
	Prevalence rate (%)														
Falls (unadjusted)	57.1	47.6	50.3	51.1	48.7	52.3	41.7	40.5	52.4	48.3	47.8	48.1	50.3	49.6	16.6
Intense or disruptive Daily Pain	41.9	44.9	42.9	47.6	46	49.5	48.8	47.4	45.8	49.5	45.9	47	45.8	52.1	10.2
Caregiver Distress	39.9	34.8	45.2	40.7	39.2	35.7	40	39.8	46.5	37.3	47.1	44.5	44.2	56.3	21.5
Negative Mood	21.4	28.9	22.3	22.6	32.4	25.1	30.4	33.5	29.5	25.3	25.5	22	27.9	27.2	12.1
Inadequate mediation to control pain	17.7	24.3	21.8	23.3	25.6	28.7	22.4	20.7	23.4	22.1	24.7	21.2	20.6	27.8	8.1
Social Isolation	19.2	21.8	16.2	17.7	20.6	20.8	25	16.6	24.1	20.8	23.7	19.8	29.9	27	13.7
Constipation	4	3.9	2.5	3.4	5.4	3.1	3.7	4.8	2.8	4.1	1.7	2.9	3.5	4.3	3.7

^a^ difference between the highest and lowest rate across the 14 geographic regions

The QI rates were stratified by four diagnoses, namely, cancer, congestive heart failure, chronic obstructive pulmonary disease/asthma/emphysema (a single item on the RAI-HC), and dementia (either Alzheimer’s dementia or any other form of dementia). These groups were not mutually exclusive, therefore an individual with multiple diagnoses could populate multiple groups (Table 4). The largest absolute differences in QI rates were seen between those who had a diagnosis of cancer and those with a diagnosis of dementia. The rate of the caregiver distress QI was higher among those with some form of dementia (52.6% vs. 32.2%), as was the rate of the falls QI (53.3% vs. 40.2%). Conversely, those with cancer were more likely to experience the pain QI than those with dementia (51.6% vs. 39.3%) (Table 4).

**Table 4:** Quality indicator rates stratified by diagnosis

Quality Indicator	Diagnosis			
	Cancer (*n* = 4580)	Congestive Heart Failure (*n* = 3657)	COPD/Emphysema/ Asthma (*n* = 3934)	Dementia (*n* = 4991)
	% (*n*)			
Prevalence of Falls (unadjusted)	40.2 (1735)	50.2 (1708)	48.2 (1781)	53.3 (2380)
Prevalence of Disruptive or Intense Daily Pain	51.6 (2363)	52.1 (1904)	50.6 (1990)	39.3 (1962)
Prevalence of Caregiver Distress	32.2 (1476)	41.5 (1519)	41.6 (1636)	52.6 (2623)
Prevalence of Negative Mood	25.9 (1187)	27.0 (986)	28.1 (1106)	23.7 (1185)
Prevalence of Inadequate Medication to Control Pain	23.1 (935)	24.0 (786)	23.7 (829)	17.5 (759)
Prevalence of Social Isolation	26.4 (1207)	28.0 (1023)	29.7 (1167)	20.3 (1015)
Prevalence of Constipation	4.4 (203)	2.8 (103)	3.1 (120)	2.9 (145)

## Discussion

To our knowledge, this is the first paper to develop QIs for seriously ill home care clients using interRAI data. These preliminary QIs have operational definitions, target those with and without cancer, and are based on items within the RAI-HC, the standardized assessment mandated in Ontario and in other parts of Canada. Three of the seven QIs were experienced by over 40% of seriously ill individuals; 42% of caregivers experiencing distress, nearly 50% of clients having falls and 47% with significant pain. All of these QIs represent undesirable outcomes that are amenable to change.

The seven QIs reported here are mainly symptom focused and would be considered outcomes of care, versus measures of the structure or the process of care. Although all three types of indicators are recommended as part of a comprehensive assessment of quality [46], most QI development efforts have focussed on process indicators [8, 47] and almost no QIs have been developed explicitly for home-based palliative care [47], a definite advantage of the current project. The QIs we have developed would fall under two key domains, namely the physical aspects of care (e.g., pain, constipation) and psychosocial aspects of care (e.g., social isolation, caregiver distress). These domains have been highlighted by multiple groups as being instrumental when attempting to assess the quality of palliative care [2, 8, 48].

Several QI rates in the current study were similar to previous research examining palliative care in the community. For example, this study found a rate of daily pain that was only slightly lower than that reported by Potter et al. (56% vs. 46%) and the rate of negative mood was nearly identical [49]. The prevalence of constipation was significantly higher in the previous study (35% vs. 3.5%) [49], which likely reflects the fact that 95% of their sample had cancer and would have a higher rate of opioid use to treat pain. This may relate to the coding of the item on the RAI-HC, which captures instances of constipation only within the last 3 days. Similarly, caregiver distress was lower in previous research (24% vs. 41% in this study) that targeted palliative home care clients, the vast majority of whom had cancer [50]. One possible explanation is that caregivers of those with other life-limiting illnesses are experiencing high rates of distress. For example, substantial evidence points to caregivers of individuals with dementia experiencing poorer self-rated health, having a greater risk of health problems and elevated levels of stress hormones compared with other caregivers [51]. This finding is supported by our results showing that caregivers of patients with dementia were more likely to experience distress than those caring for someone with cancer (42% vs. 32%).

Although some quality measures exist for several key outcomes (e.g., pain, caregiver distress, and depression) [52], there is a serious lack of specific information targeting patients receiving palliative care in their own homes, especially for QIs related to social isolation and falls. Although targeting a somewhat different population, a recent hospital-based study of palliative patients reported rates of social isolation ranging from 23 to 38%, depending on the diagnosis [53], which is in line with our findings. The rate of falling among a general population of home care clients was 34% [39], somewhat lower than that reported in this study (49%), which likely reflects the fact that our seriously ill sample were more physically and cognitively impaired.

The seriously ill group was significantly more impaired than the comparison group on the health index scales and were more likely to have been diagnosed with life-limiting illnesses. This group also experienced higher rates (absolute differences between 3 and 15%) of inadequate pain control, daily pain, falls, social isolation, and negative mood when compared to a general home care population aged 65+ in Ontario [39]. The results reported here support the methodology chosen to identify a group who were seriously ill and could potentially benefit from a palliative approach to care. This choice of target population is supported by other research showing that identifying the need for palliative care is essential for those with less predictable or non-cancer diagnoses [5]. To effectively identify the need for palliative care, it is critical to look at factors beyond prognosis, as recommended by organizations such as the Canadian Hospice Palliative Care Association, the World Health Organization and the International Association for Hospice and Palliative care [54–56].

This study was limited to using the items available within the RAI-HC and, as a result, some of the suggestions from the expert panel could not be accommodated and therefore we could not develop all of the QIs suggested. The proposed QIs focus mainly on the outcomes of care, partly because those were the suggestions made by the experts, and also since outcomes are more easily measured with the RAI-HC. The experts had very few recommendations for QIs related to the process or structure of care, limiting our capacity to make causal links between these various aspects of care [57]. Another limitation is the fact that consultations did not include other key stakeholders such as policy-makers, clients, and their families. This will be important to address in future projects to ensure that the QIs fully capture the salient issues from multiple groups. Finally, we were unable to determine whether these clients were receiving palliative home care services. As such, our results apply only to seriously ill clients and the results may be different had we limited our sample to individuals on a palliative caseload and known to be receiving palliative care services.

In Ontario, several initiatives are underway to improve the quality of palliative care services across the province. For example, Health Quality Ontario and the Ontario Palliative Care Network are developing quality standards and indicators for palliative services [58, 59]. The preliminary list of QIs proposed here can make a significant contribution to this process as these organizations work collaboratively with home care providers and researchers to establish a set of standardized measures. The potential QIs, based on the RAI-HC, can serve as decision-support tools during the process of continuous quality improvement, while organizations attempt to understand the current quality issues they face and how interventions can influence the QI rates over time.

## Conclusion

Home care clients in Ontario who are seriously ill are experiencing high rates of negative health outcomes, many of which are amenable to change. The RAI-HC can be a useful tool in identify these clients in order to better understand their needs and abilities. As demonstrated here, the RAI-HC can also be used to flag potential quality issues at the organizational level. Although only the first step in a larger program of research to develop standardized QIs, these results contribute significantly to the process of creating and validating a standardized set of QIs that can be generated by organizations using the RAI-HC as part of normal clinical practice locally and nationally.

## Abbreviations

ADL-S: Activities of daily living self-performance hierarchy scale
CHESS: Changes in health end-state signs and symptoms
CI: Confidence interval
CPS: Cognitive performance scale
DRS: Depression rating scale
IADLs: Instrumental activities of daily living
OR: Odds ratio
QIs: Quality indicators
RAI-HC: Resident assessment instrument for home care
